# Treatment of Rhenium-Containing Effluents Using Environmentally Friendly Sorbent, *Saccharomyces cerevisiae* Biomass

**DOI:** 10.3390/ma14164763

**Published:** 2021-08-23

**Authors:** Inga Zinicovscaia, Nikita Yushin, Dmitrii Grozdov, Konstantin Vergel, Pavel Nekhoroshkov, Elena Rodlovskaya

**Affiliations:** 1 Department of Nuclear Physics, Joint Institute for Nuclear Research, Joliot-Curie Str., 6, 1419890 Dubna, Russia; ynik_62@mail.ru (N.Y.); dsgrozdov@rambler.ru (D.G.); verkn@mail.ru (K.V.); p.nekhoroshkov@gmail.com (P.N.); 2 Department of Nuclear Physics, Horia Hulubei National Institute for R&D in Physics and Nuclear Engineering, 30 Reactorului Str. MG-6, 077125 Magurele, Romania; 3 Laboratory for Heterochain Polymers, A.N. Nesmeyanov Institute of Organoelement Compounds of Russian Academy of Sciences, Vavilova Str., 28, 119991 Moscow, Russia; ro745@mail.ru

**Keywords:** biosorption, rhenium, copper, molybdenum, yeast

## Abstract

Yeast *Saccharomyces cerevisiae* biomass was applied for rhenium and accompanying elements (copper and molybdenum) removal from single- and multi-component systems (Re, Re-Mo, Re-Cu, and Re-Mo-Cu). Yeast biomass was characterized using X-ray diffraction, scanning electron microscopy, and Fourier transform infrared spectroscopy. The effects of biosorption experimental parameters such as solution pH (2.0–6.0), rhenium concentration (10–100 mg/L), time of interaction (5–120 min), and temperature (20–50 °C) have been discussed in detail. Maximum removal of rhenium (75–84%) and molybdenum (85%) was attained at pH 2.0, while pH 3.0–5.0 was more favorable for copper ions removal (53–68%). The Langmuir, Freundlich, and Temkin isotherm models were used to describe the equilibrium sorption of rhenium on yeast biomass. Langmuir isotherm shows the maximum yeast adsorption capacities toward rhenium ions ranged between 7.7 and 33 mg/g. Several kinetic models (pseudo-first-order, pseudo-second-order, and Elovich) were applied to define the best correlation for each metal. Biosorption of metal ions was well-fitted by Elovich and pseudo-first-order models. The negative free energy reflected the feasibility and spontaneous nature of the biosorption process. *Saccharomyces cerevisiae* biomass can be considered as a perspective biosorbent for metal removal.

## 1. Introduction

Rhenium, for which world production and consumption are at a level of 60 tons per year, is one of the rarest elements on Earth [1,2,3]. Nevertheless, unique physical and chemical characteristics make rhenium a critical metal of high industrial importance. Presently, rhenium is applied in the aerospace industry, production of alloys, electromagnets, catalysts, semiconductors, thermocouples, heating elements, metallic coatings, vacuum tubes, X-ray tubes, and medical devices [2,3,4]. Rhenium does not form its own deposits. Increased concentrations of rhenium can be found in copper and molybdenum deposits, and it also occurs as an isomorphic impurity in more than 50 carrier minerals [1,3]. In industry, 80% of sources of rhenium raw materials are molybdenum and copper sulfide concentrates, produced by Chile, Kazakhstan, France, Germany, Russia, the U.S., China, Great Britain, the Netherlands, and Poland [1,5]. The techniques of rhenium extraction involve removing Re_2_O_7_ from the sulfurous gas phase generated during hearth roasting or smelting [4]. During the process of extraction, part of rhenium is dispersed as volatile Re_2_O_7_ in soils and as ReO_4_^−^ions in industrial effluents and water [5]. Anthropogenic sources of rhenium emission in the environment also include mining, copper and molybdenum ore processing, motorways, coal-burning plants, non-ferrous metal smelters, and scrap recycling units [3].

Several traditional techniques including chemical deposition, sorption, ion exchange, capillary electrophoresis, liquid chromatography, and solvent extraction are used for rhenium recovery from industrial effluents. Rhenium is preferentially recovered using strongly basic anion exchangers [6]. The main drawbacks of the mentioned techniques are associated with high operational cost, low removal efficiency when metal is present in solution in low concentration, and the possibility of toxic sludge generation leading to recontamination of water, which requires additional treatment [7]. Some techniques, including extraction, are dangerous because of the use of a flammable organic phase [8].

These drawbacks forced researchers to find economically profitable and safe methods for rhenium effluents treatment. Sorption, which has proven to be one of the most effective methods for the removal of toxic metals, is widely used for rhenium recovery from aqueous solutions [9,10]. Among several types of sorption processes, biosorption is one of the most promising owing to the availability, high efficiency, and low costs of biosorbents [10].

Information about the application of biological sorbents for rhenium removal is very limited. In our previous work, the microalga *Spirulina platensis* was used for rhenium removal from a single-component batch system and two rhenium-containing industrial effluents [11]. Mashkani and co-authors [12] tested more than 100 bacterial strains, and only *Bacillus* sp. GT-83 showed good absorption capacity for rhenium. Xiong et al. [13] applied brown algae *Laminaria japonica* chemically modified with sulfuric acid for rhenium biosorption.

Yeast *Saccharomyces cerevisiae* is one of the microorganisms, which is often studied as a model object in bioremediation experiments. Applicability of *Saccharomyces cerevisiae* for metal ions biosorption is attributed to ease of its cultivation on a large scale, use of cheap media, high biomass production, safety, and high biosorption capacity. *Saccharomyces cerevisiae* biomass is obtained in large amounts as a byproduct of the brewing and food industries, and its use remains largely unexploited; therefore, its disposal is frequently an environmental problem [14,15,16].

In the present study, the ability of *Saccharomyces cerevisiae* biomass to treat rhenium-containing effluents was investigated as a function of initial solution pH, time of sorbent contact with sorbate, rhenium concentration, and temperature. Different equilibrium and kinetic adsorption models were applied to the experimental data to find out the best fit. The thermodynamics of the sorption are discussed.

## 2. Materials and Methods

### 2.1. Batch Solutions

The metal solutions with given concentrations were prepared by dissolving a suitable amount of NaReO_4_, CuSO_4_, and Na_2_MoO_4_ (Sigma Aldrich, analytical grade) in deionized water. Since rhenium is mainly recovered as a byproduct of molybdenum and copper processing, four systems with the following composition were prepared: Re, Re-Mo, Re-Cu, and Re-Cu-Mo. The concentration of rhenium, as an element of main interest, in all systems was 10 mg/L, and the concentration of copper and molybdenum was 5 mg/L.

### 2.2. Biosorbent 

Yeast Saccharomyces cerevisiae biomass used in experiments was obtained from residues generated by a brewing company (Chisinau, Republic of Moldova). The procedure of biosorbent preparation is presented in detail in our previous study [17,18]. In particular, biomass was dried at 105 °C and homogenized before analysis.

### 2.3. Biosorption Procedure

Metal ions biosorption was investigated over a pH range of 2.0–6.0, time 5–120 min, rhenium concentration 10–100 mg/L, and temperature 20–50 °C. The batch experiments were initiated by mixing 500 mg of dried yeast biomass with 50 mL of experimental solution, which was shaken continuously at 200 rpm for defined intervals of time. The isotherm, kinetic, and thermodynamic experiments were performed according to the same scheme, varying parameters of interest (time, concentration, or temperature) and keeping other parameters constant. At the end of the experiments, biomass was separated from the solution by filtration, dried at 105 °C, weighted at analytical balance, and packed in aluminum foil cups for neutron activation analysis. Copper concentration in the solution was measured using atomic absorption spectrometry. All experiments were performed in duplicate. The scheme of biosorption experiment is presented in (Supplementary Materials Figure S1).

### 2.4. Data Processing

Adsorption capacity (q, mg/g) and removal efficiency (E,%) were calculated using the following Equations (1) and (2):

(1) $$q=\frac{V\left(C_{i}-C_{f}\right)}{m}$$

(2) $$E=\frac{C_{i}-C_{f}}{C_{i}}\times 100$$

where *q* is the amount of metal ions adsorbed on the sorbent (mg/g), *V* is the volume of solution used (L), *C_i_* and *C_f_* are the initial and final metal concentrations in the solution (mg/L), and m is the mass of sorbent (g).

The thermodynamic parameters (Gibbs energy (ΔG°), enthalpy (ΔH°), and entropy (ΔS°)) values were calculated using Equations (3)–(5):

(3) $$\ln K_{d}=\frac{\Delta S^{0}}{R}-\frac{\Delta H^{0}}{RT}$$

(4) ΔG°=ΔH°−TΔS°

where *K_d_* is the distribution coefficient and is calculated according to Equation (13):

(5) $$K_{d}=\frac{\left(C_{0}-C_{e}\right)V}{mC_{e}}$$

where *C*_0_ is the initial concentration of metal ions (mg/L), *C_e_* is equilibrium metal ion concentration (mg/L), *V* is the volume of solution (L), and *m* is biosorbent mass (g).

Pseudo-first-order (6), pseudo-second-order (7), and Elovich models (8) were applied to describe the kinetics of the metal sorption on yeast biomass. Non-linear regression was used to estimate parameters of adsorption, since it allows minimizing the error distribution between the experimental and predicted data [11].

(6) $$q_{t}=q_{e\;}\left(1-e^{-k_{1}t}\right)$$

(7) $$q=\frac{q_{e}^{2}k_{2}t}{1+q_{e}k_{2}t}$$

(8) $$q_{t\;}=\frac{1}{\beta }\ln\left(1+\alpha \beta t\right)$$

In these models, *q_e_* and *q_t_* are the quantities of metal (mg/g) sorbed from the solution at equilibrium and at *t* (min) time, respectively; *k*_1_ (1/min)/*k*_2_ (g/mg·min) are the pseudo-first-order/second-order rate constants; and *α* (g/mg∙min) and *β* (g/mg) are Elovich equation constants.

Equilibrium data were fitted using non-linearized Langmuir, Freundlich, and Temkin isotherm models (9)–(11):

(9) $$q_{e}=\frac{q_{m\;}bC_{e}}{1+bC_{e}}$$

(10) $$q_{e}=K_{F}Ce^{\frac{1}{n}}$$

(11) $$q_{e}=\frac{RT}{b_{T}}\ln\left(a_{T}C_{e}\right)$$

where *C_e_* is equilibrium concentration (mg/L), *q_m_* is maximum adsorption capacity (mg/g), *b* is Langmuir adsorption constant (L/mg), *K_F_* and *n* are Freundlich equation constants, 1/*b_T_* manifests the sorption potential of the sorbent, *a_T_* is Temkin constant, *R* is the universal gas constant (8.314 J K^−1^ mol^–1^), and *T* is the temperature (K) [11,13].

Separation factor *R_L_*, which predicts the potential adsorption probability relationship between solid and liquid, was calculated according to Equation (12).

(12) $$R_{L}=\frac{1}{1+bC_{0}}$$

where 0 < *R_L_* < 1 indicates favorable adsorption, *R_L_* > 1—unfavorable adsorption, *R_L_* = 1—linear adsorption, and *R_L_* = 0—irreversible adsorption [13].

### 2.5. Methods

The concentration of rhenium and molybdenum adsorbed by yeast biomass was determined by neutron activation analysis at the IBR-2 reactor (Dubna, Russia). The procedure of sample preparation for analysis, main parameters of sample irradiation, and data processing are presented in detail elsewhere [16,17,18]. Samples, packed in aluminum cups, were irradiated at a neutron flux of 1.2 × 10^12^ cm^−2^ s^−1^ for 72 h, re-packed, and measured for 30 min. Induced gamma activity was measured using HP-Ge detectors, and gamma spectra processing was performed using Genie 2000. The determination of the content of elements was done using software developed in the Sector of Neutron Activation Analysis and Applied Research. Copper concentration in solution was measured using atomic absorption spectrometer Thermo Scientific iCE 3400 (Thermo Fisher Scientific, Waltham, MA, USA) with electrothermal atomization.

Yeast surface analysis was performed using the S3400N scanning electron microscope (Hitachi, New York, NJ, USA). For measurement, yeast samples were placed on the aluminum stub and coated with palladium in real time for 100 seconds using an MC1000 Ion Sputter Coater (Hitachi, USA). X-ray diffraction patterns (XRD) of the yeast biomass were carried out on an X-ray diffractometer EMPYREAN (PANalytical, Malvern, UK) in Cu-Kα radiation λ = 1.541874 Å. Infrared spectra were recorded in the range of 4000–400 cm^−1^ using a Bruker Alpha Platinum-ATR spectrometer (Bruker Optics, Ettingen, Germany).

## 3. Results and Discussion

### 3.1. Adsorbents Characterization

**Figure 1:**
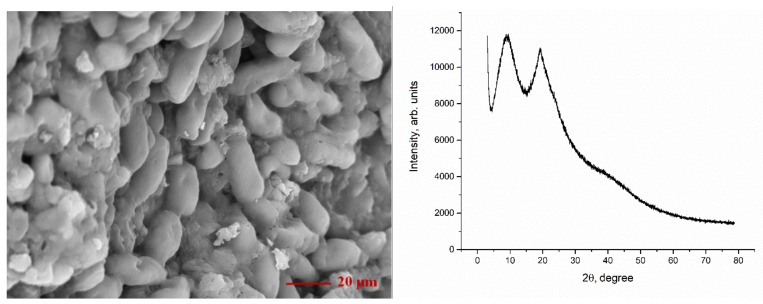
SEM image (**left**) and XRD pattern (**right**) of S. cerevisiae yeast biomass before biosorption experiments.

Scanning electron microscopy (SEM) was applied to provide high-resolution images from the surface of the biosorbent. According to Figure 1 yeast, S. cerevisiae had a smooth cell surface. Yeast cells are oval and immobilized close to each other [18,19]. It should be mentioned that after biosorption process, the surface of the biosorbent did not undergo significant modification.

In the XRD spectrum (Figure 1), the broad peak around 2θ = 200 corresponds to the amorphous phase of biomass, which is in agreement with already published results [18,19]. This is expected, since biomass is composed of an inactivated microorganism and organic residues [20]. 

### 3.2. Solution Acidity

In the biosorption experiments, the efficiency of metal removal depends heavily on the pH of the solution, since it controls the charge of the biosorbent surface and metal specification. In the present study, the pH of experimental solutions changed from 2.0 to 6.0, and the time of interaction was 1 h. The pH was adjusted to desired values by the addition of NaOH or HNO_3_. The difference in the pH values of solutions before and after biosorption experiments was in the range of 0.1–0.2. 

**Figure 2:**
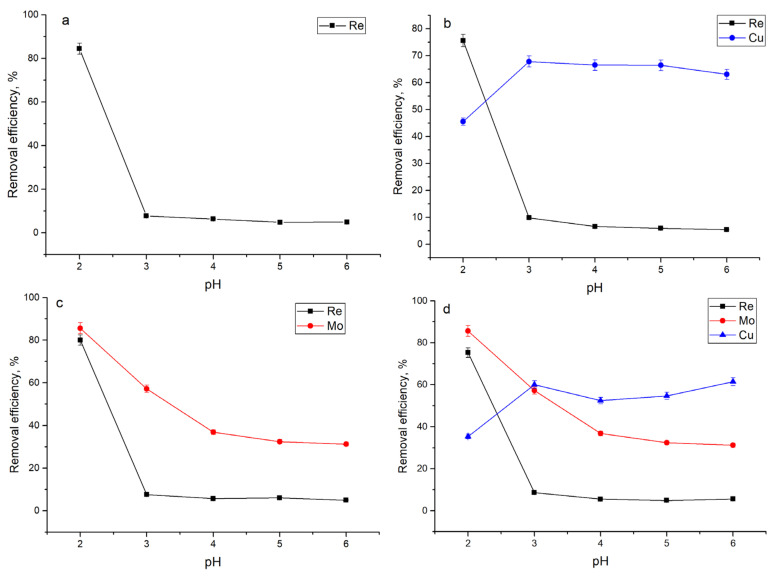
Effect of initial solution pH on the adsorption of metal ions present in analyzed systems: (**a**) Re, (**b**) Re-Cu, (**c**) Re-Mo, and (**d**) Re-Mo-Cu.

Maximum rhenium removal (Figure 2) in all analyzed systems was achieved at pH 2.0: 84% in Re system, 76% in Re-Cu system, 80% in Re-Mo system, and 75% in Re-Cu-Mo system. With the increase in the pH values, the efficiency of rhenium removal significantly decreased, reaching 5% at pH 6.0. Such behavior of rhenium removal is in agreement with our previous study, where cyanobacteria *Spirulina platensis* was used as a biosorbent [11]. Maximum sorption of rhenium on *Bacillus* sp. GT-83 was obtained at pH = 2.5 [12]. The high efficiency of rhenium removal at pH 2.0 can be explained by the preference of the positively charged surface groups for negatively charged metal ion species binding. Within the studied pH range, ReO_4_^−^ is the dominant rhenium species in the solution [21]. With the increase in pH, the surface of the yeast biomass becomes negatively charged, impeding rhenium ions removal [18,22]. 

Along with rhenium, molybdenum ions were better sorbed from solution at low pH values. The highest efficiency of molybdenum removal in both Re-Mo and Re-Mo-Cu systems was 85%. Molybdenum in the analyzed complex system is present in solution in the dissolved form from pH 2.0–3.0. In the pH range 3.0–6.0, molybdenum is present in the form of copper molybdate [17] that can explain the decrease in the efficiency of its removal. A decrease in molybdenum sorption with an increase in pH was also shown for other sorbents [17,23,24]. 

Removal of copper was more favorable at pH 3.0–5.0 in the Re-Cu system (66–68%) and pH 6.0 in the Re-Mo-Cu system. However, it should be mentioned that at pH range 3.0–6.0, copper removal was relatively high, at 53–61%. Maximum copper removal from multimetal systems by *S. cerevisiae* was achieved at pH 3.0 [25] and from a single component system and industrial effluent at pH 5.0 [15,26]. 

### 3.3. Thermodynamic Studies

**Figure 3:**
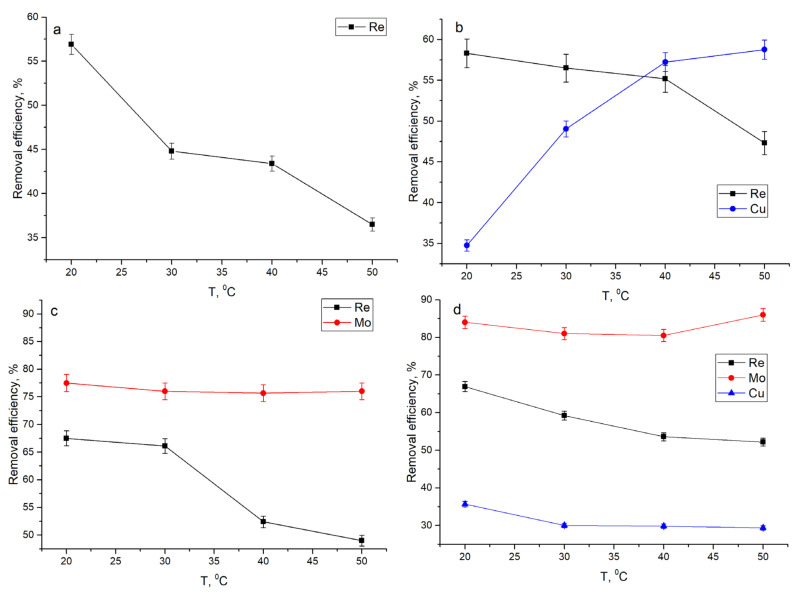
Effect of temperature on metal ions removal from analyzed systems: (**a**) Re, (**b**) Re-Cu, (**c**) Re-Mo, and (**d**) Re-Mo-Cu.

Temperature can greatly affect the efficiency of metal ions removal. Increase in temperature from 20 to 50 °C (Figure 3) lead to the decrease in rhenium removal in all analyzed systems in by 11–20% on average. The most relevant decline was noticed in Re system (by 20%). The decrease in the rhenium removal capacity with the increase in temperature can be explained by the desorption of rhenium ions from the biosorbent surface and indicates that biosorption of rhenium ions on yeast biomass is an exothermic process [27]. 

Copper removal in the Re-Cu system was favored by the increase in the temperature, which resulted in the increase in its removal from 35 to 59%, indicating that the removal process was endothermic. The efficiency of copper removal in the Re-Mo-Cu system did not depend on the temperature change. Removal of molybdenum in both analyzed systems was not affected by temperature increase (Figure 3).

The values of ΔH° and ΔS°, calculated from the slope and intercept of the plot of lnK_d_ versus 1/T (Supplementary Materials Figure S2), respectively), and ΔG° are summarized in Table 1.

The negative ΔG° values obtained for all elements point at the feasibility and spontaneous nature of the biosorption. According to enthalpy values, the biosorption of rhenium in all systems was exothermic. The positive values of ΔS° indicate the irreversibility and stability of the biosorption process [28] for elements in Re-Cu and Re-Mo systems. The same pattern was observed for molybdenum Re-Mo-Cu systems. The negative ΔS° value obtained for rhenium in Re- and Re-Mo-Cu systems, as well as copper in Re-Mo-Cu systems, point to the decrease in the randomness of the system during biosorption [29]. The positive enthalpy change for copper in the Re-Cu system and molybdenum in the Re-Mo-Cu system indicates an endothermic adsorption process. Based on ΔH° values, biosorption of all elements, except molybdenum in the Re-Mo-Cu system, was driven by a physisorption process, and for molybdenum, the sorption was chemical in nature. 

### 3.4. Kinetic Studies

**Figure 4:**
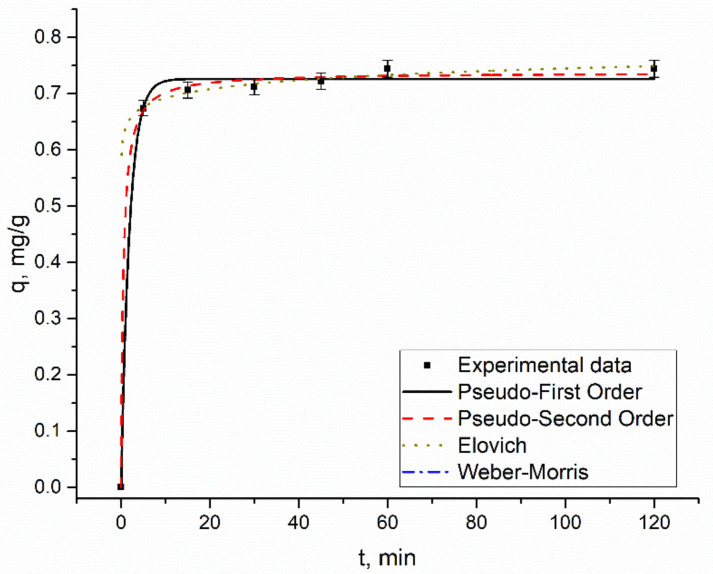
Kinetics of rhenium biosorption on yeast biomass.

**Figure 5:**
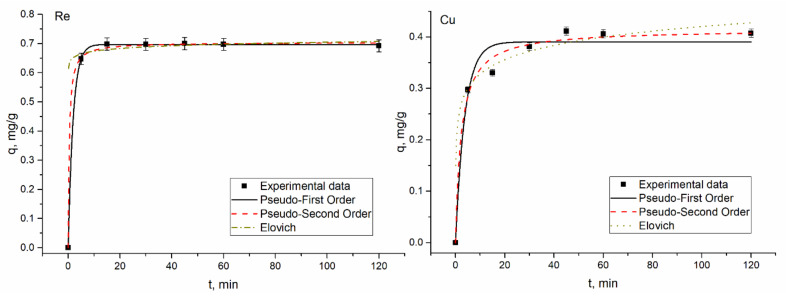
Kinetics of rhenium and copper biosorption on yeast biomass.

**Figure 6:**
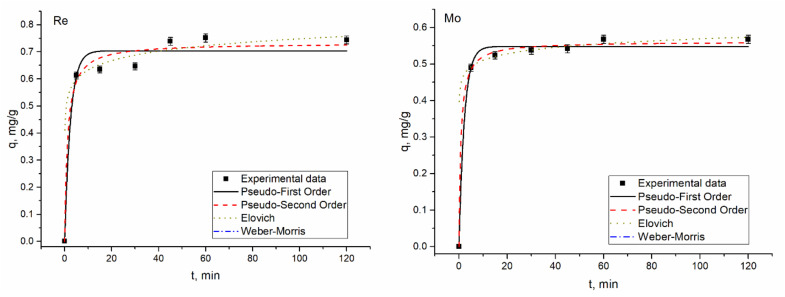
Kinetics of rhenium and copper biosorption on yeast biomass.

**Figure 7:**
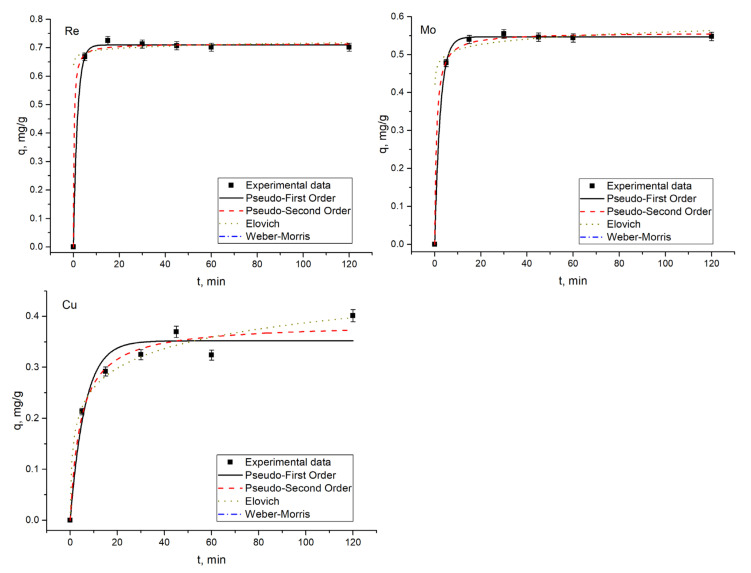
Kinetics of rhenium and copper biosorption on yeast biomass.

The time required for maximum removal of metal ions plays a critical role in the adsorption process. According to Figure 4, Figure 5, Figure 6 and Figure 7, a considerable percentage of metal ions was removed in the first 5 min of sorbent interaction with sorbate. The maximum percentage of removal in analyzed systems ranged from 70–75% for rhenium in 45–60 min, 90–94% for molybdenum in 45 min, and 45–46% for copper in 45 min. Equilibrium was reached after the contact period mentioned. High removal efficiency in the first minutes of interaction is explained by the availability of a large number of binding sites on the sorbent surface. As time proceeds, the removal decreases due to the occupation of the vacant sites on the surface by metal ions. For example, 68% of rhenium ions were removed from the solution in 5 min, and in 60 min equilibrium was achieved. However, the removal efficiency of sorbent increased by only 7%. Therefore, further increases in contact time did not enhance the biosorption process. The same pattern was observed for other analyzed systems. The low efficiency of copper removal is attributed to low pH values at which kinetic experiments were performed. 

In adsorption study, isotherms and kinetics of the sorption process provide important information underlying the mechanisms and dynamics of the process [30]. Thus, adsorption kinetic studies provide information on the adsorption rate, the performance of the adsorbent used, and the mechanism of metal sorption [31]. Many kinetic models were developed to find intrinsic kinetic adsorption constants [30]. Experimental and predicted values of biosorption capacity and other parameters of applied kinetic models are presented in Table 2. 

Usually, the coefficient of determination (R^2^) is used to determine which model better describes metal sorption. According to Table 2, R^2^ values for all applied models are very high, ranging from 0.94–0.99, showing that the three kinetic models present a good fit to the experimental data. Another consideration was based on a comparison of the theoretical and experimental values of biosorption capacity in pseudo-first-order and pseudo-second-order models. Experimental values of adsorption capacity and the calculated ones were in good agreement. Finally, the Akaike Information Criterion (AIC) test was applied to estimate which model is more suitable for the description of the experimentally obtained values. When comparing two fitting models, the one with the smaller AIC value is considered to be a better model. According to the AIC test metal ions, biosorption in Re-Cu and Re-Mo systems is adequately represented by the Elovich kinetic model. The model was also applicable for copper biosorption on yeast in the Re-Mo-Cu system. The Elovich equation describes the predominantly chemical sorption on highly heterogeneous sorbents [16,32]. Biosorption of rhenium in Re and Re-Mo-Cu systems, as well as of molybdenum in Re-Mo-Cu systems, was defined by the pseudo-first-order model, which suggests one-site-occupancy adsorption [16]. 

Adsorption of rhenium ions on nano-Al_2_O_3_ [8], *Laminaria japonica* chemically modified with sulfuric acid [13], and modified corn stalk [33] was better described by the pseudo-second-order model.

From the kinetics data, the activation energy (E_a_) for the binding metal ions was determined:

$$lnk_{2}=lnA-\frac{E_{a}}{RT}$$

where *E_a_* is the Arrhenius activation energy of adsorption, *A* is the Arrhenius factor, *R* is the gas constant, and *T* is the solution temperature.

Since the *E_a_* values for all elements (Table 2) were less than 40 kJ/mol, physical sorption may play an important role in metal ions removal. 

### 3.5. Equilibrium Studies 

The effect of rhenium concentration on the yeast biosorption capacity was investigated by changing its concentration in solution from 10 to 100 mg/L, and the concentration of copper and molybdenum in solution was maintained as constant. An increase in rhenium concentration in solution promotes its sorption by yeast biomass. In three systems, namely Re, Re-Cu, and Re-Mo, biosorption capacity increased on average from 0.6 to 5.0 mg/g as the rhenium concentration rose, while in the Re-Mo-Cu system the increase in sorption capacity was lower, ranging from 0.7–2.9 mg/g. 

An increase in rhenium concentration in the Re-Cu system resulted in a decrease in copper removal by approximately 15%. Removal of molybdenum in the Re-Mo system was not affected by the rise of rhenium concentration. In the Re-Mo-Cu system, copper removal was almost the same at all rhenium concentrations, while removal of molybdenum decreased at high rhenium concentrations (75 and 100 mg/L) by approximately 15%. 

**Figure 8:**
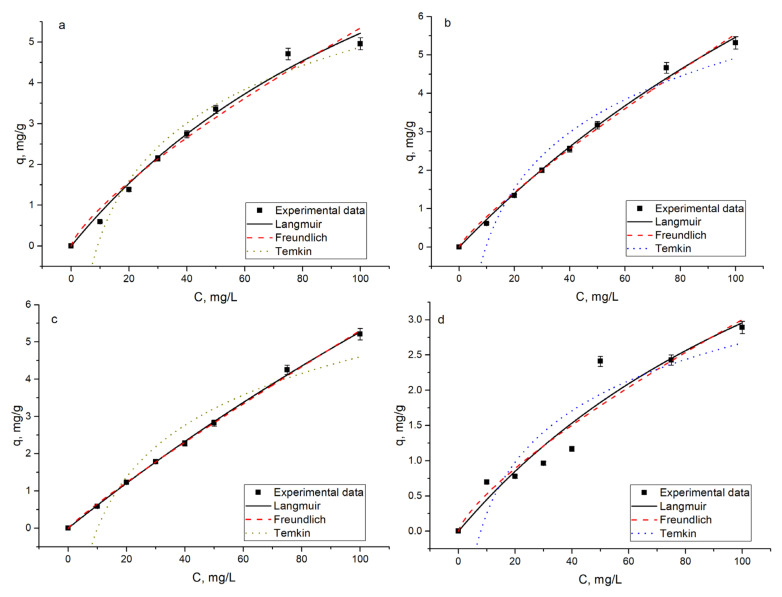
The adsorption isotherms and experimental data for rhenium ion sorption on yeast biomass. (**a**) Re, (**b**) Re-Cu, (**c**) Re-Mo, and (**d**) Re-Mo-Cu.

The parameters from the adsorption equilibrium models provide useful information on surface properties, adsorption mechanism, and interaction between the adsorbent and adsorbate [30]. The graphical representation of used models among experimental data is presented in Figure 8, and calculated models’ parameters are given in Table 3. 

The coefficient of determination values obtained for Langmuir and Freundlich models (Table 3) indicate positive evidence that the adsorption of rhenium on yeast biomass may follow these isotherms. The AIC test was applied to estimate which model better describes rhenium ions sorption. Obtained results revealed that the Langmuir model fit better for the description of experimental data, suggesting that rhenium biosorption mainly occurred by chemisorption within the monolayer [34].

The Langmuir model is usually applied to describe homogeneous adsorption, considering that all adsorption sites on the surface of a biosorbent have equal solute affinity [35]. The maximum sorption capacity obtained by using the Langmuir isotherm for all systems was significantly higher than experimentally obtained values. The Langmuir isotherm fitted well the experimental data for rhenium sorption on *Laminaria japonica* chemically modified with sulfuric acid [13].

The favorability of the biosorption process was confirmed by R_L_ values lower than 1.0. The coefficients of correlation obtained for the Temkin isotherm were lower than for the other two models, which indicates the unsuitability of the model to describe the rhenium ions biosorption on yeast biomass.

Yeast sorption capacity was compared with the values available in the literature for other types of sorbents (Table 4). The adsorption capacity of yeast biomass was lower than values obtained in works [9,11,33] and higher than in other studies presented in Table 4. However, it should be mentioned that experiments were performed with different experimental conditions, and in the main part of the previously conducted research, rhenium removal from a single-element system was studied.

### 3.6. Mechanism of Metal Ions Biosorption

**Figure 9:**
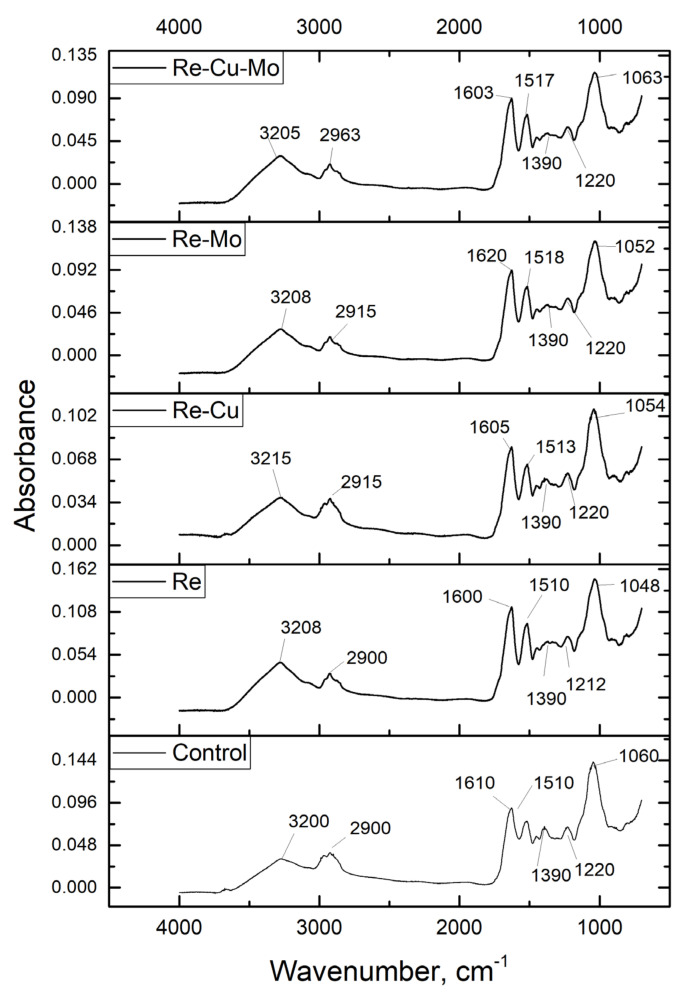
FTIR spectra of control and metal-loaded yeast biomass.

Metal removal from solution is possible through several mechanisms: metal binding to functional groups, ion exchange, physical sorption, microprecipitation, and others. To prove the involvement of functional groups in metal trapping, FTIR spectra of control and metal-loaded biomass were recorded (Figure 9).

In the control spectrum, the intensive bands at 1060 and 1510 cm^−1^ correspond to vibrations of OH groups. The peak at 1220 cm^−1^ is related to stretching vibrations of the С=О group, and the sections at 1390 and 2900 cm^−1^ correspond to the vibration of alkyl groups, either –СН_3_ or –СН_2_. The peak at 1610 cm^−1^ is attributed to vibration of unsaturated bond СН=СН. The strong peak at wavenumber area 3400–3200 cm^−1^ could be characteristic for hydroxyl (–OH) and amine (–NH) groups. In addition, –C–O, –C–C, and –C–OH stretching vibrations found at the adsorption peaks of the 1650–1200 cm^−1^ region could correspond to –C–O, –C–C, and –C–OH stretching vibrations or the amide I–III bands of polypeptide/proteins [18]. In the Re-loaded spectrum, the shift of bands 1048, 1212, 1600 and 3208 cm^−1^ indicates involvement of ОН, СН=СН, and amine groups in rhenium binding. At acidic pH, a large number of amine groups are in the protonated form and can interact with the negatively charged perrhenate anion ReO_4_^−^ via electrostatic interactions [20]. Participation of amine groups in rhenium sorption by Spirulina platensis was shown in our previous study [11]. OH– and –NH_2_ participated in rhenium removal by brown alga Laminaria japonica [13]. Zhang and coauthors [8] also suggest an important role of hydroxy groups in rhenium removal.

In the Re-Cu system, in addition to ОН, СН=СН and NН_2_ groups, which play important roles in rhenium removal, a shift of the band of alkyl groups by 15 cm^−1^ took place. As was shown in isotherms studies, an increase in rhenium concentration in solution did not affect significantly the removal of copper ions, which can be explained by the preference of rhenium and copper for different functional groups. Hydroxyl and carbonyl groups play important role in copper ions removal by yeast biomass [20]. The shift of the bands of the same groups was observed in the Re-Mo system. Rhenium and molybdenum have similar chemical properties, so it can be suggested that they bind to the same groups on the biosorbent surface. However, it should be mentioned that in performed experiments, rhenium removal efficiency was not affected by the presence of molybdenum ions in solution. According to Shan et al. [36], molybdenum ions have a high affinity to the alkyl group on the N-atom of the amine group and steric hindrance to the amine group. In the Re-Cu-Mo system, amine, hydroxyl and alkyl groups participated in removal. The decrease in molybdenum removal in equilibrium experiments can be explained by its competition with copper ions for the same binding sites.

## 4. Conclusions

Yeast *Saccharomyces cerevisiae* biomass was applied to remove elements from rhenium-containing effluents. The experimental results indicate the high efficiency of rhenium and molybdenum removal (up to 86%) at pH 2.0 and of copper at pH range 3.0–5.0 (up to 68%). The equilibrium data were well fitted by the Langmuir adsorption isotherm equations, with a maximum adsorption capacity in the range of 7.7–33 mg/kg. The highest amount of metals was removed from solutions in the first 5 min of sorbent interaction with sorbate, and equilibrium was attained in 45–60 min for all systems. Metal removal from analyzed systems could be expressed well by the pseudo-first-order and Elovich models. The thermodynamic parameters indicated the feasibility and exothermic and spontaneous nature of rhenium biosorption on yeast biomass. The process of molybdenum and copper removal was a spontaneous, endothermic adsorption process. Even equilibrium and kinetic data indicated chemisorption as the main mechanism of rhenium biosorption on yeast biomass, and the thermodynamic parameters also point to the occurrence of physical absorption. FTIR data indicate the involvement of amine, hydroxyl, and alkyl groups in metal ions removal. Yeast *Saccharomyces cerevisiae* can be considered for the development of efficient and cheap biosorbent for rhenium-containing effluents treatment.

## Figures and Tables

**Table 1:** Thermodynamic parameters of metal ions biosorption on yeast biomass.

System	Metal	∆*G*^◦^, kJ/mol				∆*H*^◦^, kJ/mol	∆*S*^◦^, J/mol·K	*R* ^2^
		293 K	303 K	313 K	323 K			
Re	Re	−9.8	−9.7	−9.7	−9.6	−11.4	−5.8	0.96
Re-Cu	Re	−9.9	−10.1	−10.3	−10.4	−5.05	16.6	0.89
	Cu	−9.3	−9.7	−10.1	−10.5	2.6	41	0.99
Re-Mo	Re	−10.3	−10.4	−10.4	−10.4	−9.3	3.3	0.94
	Mo	−10.5	−10.8	−11.1	−11.5	−0.7	33	0.92
Re-Mo-Cu	Re	−16.0	−15.2	−14.2	−13.6	−39.4	−79.8	0.96
	Mo	−18.9	−19.7	−20.5	−21.3	46.7	80.6	0.74
	Cu	−11.4	−11.3	−11.3	−11.3	−12.5	−3.1	0.84

**Table 2:** Parameters of the kinetic models applied to investigate metal sorption on yeast biomass.

Model		Re	Re-Cu		Re-Mo		Re-Mo-Cu		
	Metal	Re	Re	Cu	Re	Mo	Re	Mo	Cu
PFO *	q_exp_, mg/g	0.74	0.69	0.39	0.74	0.57	0.7	0.54	0.37
	q_e_,_cal_, mg/g	0.72	0.7	0.4	0.7	0.55	0.7	0.55	0.35
	k_1_, min^−1^	0.52	0.05	0.26	0.4	0.44	0.57	0.41	0.16
	R^2^	**0.99**	0.99	0.96	0.96	0.99	**0.99**	**0.99**	0.94
PSO	q_e, cal_, mg/g	0.74	0.7	0.41	0.73	0.56	0.71	0.56	0.39
	k_2_, g/mg·min	2.7	3.5	1.05	1.1	2.2	4.5	2.3	0.5
	R^2^	0.99	0.99	0.99	0.98	0.99	0.99	0.99	0.97
	Ea, kJ/mol	2.2	2.8	0.12	0.2	1.9	3.7	2.0	1.7
EM	α, mg/g·min	3.3	1.5	1.4	1.6	1.2	8.8	1.8	0.6
	β, g/min	43.8	75.2	24.8	19.9	39.1	89	49	0.18
	R^2^	0.99	**0.99**	**0.99**	**0.99**	**0.99**	0.99	0.99	**0.98**

* PFO—pseudo-first-order model; PSO—pseudo-second-order-model; EM—Elovich model.

**Table 3:** Langmuir, Freundlich, and Temkin isotherms parameters for the sorption of rhenium on yeast biomass.

Model	Parameters	Re	Re-Cu	Re-Mo	Re-Mo-Cu
Langmuir	q_m_, mg/g	13	20	33	7.7
	b, L/mg	0.007	0.004	0.002	0.007
	R_L_	0.58–0.98	0.71–0.96	0.83–0.98	0.58–0.98
	R^2^	**0.99**	**0.99**	**0.99**	**0.90**
Freundlich	K_F_, mg/g	0.16	0.11	0.08	0.09
	1/n	0.76	0.79	0.9	0.75
	R^2^	0.97	0.99	0.99	0.90
Temkin	a_T_, L/g	0.11	0.1	0.099	0.13
	b_T_, kJ/mol	1.2	1.15	1.2	2.3
	R^2^	0.97	0.95	0.93	0.84

**Table 4:** The comparison of sorption capacity of yeast biomass sorbents with literature data.

Sorbent	q, mg/g	Reference
*Saccharomyces cerevisiae*	7.7–33	Present study
*Spirulina platensis*	142.9	[11]
Trialkylamine-Containing Impregnates	128	[9]
modified nano-Al_2_O_3_	1.93	[8]
Brown algae Laminaria japonica	37.2	[13]
Modified corn stalk	98.69	[33]

## Data Availability

Data sharing not available.

## Supplementary Materials

The following are available online at https://www.mdpi.com/article/10.3390/ma14164763/s1, Figure S1, The scheme of biosorption experiment. Figure S2, The linear plots of lnKd versus 1/T for the adsorption of metal ions.
