# Supplementary material for: Treatment of Rhenium-Containing Effluents Using Environmentally Friendly Sorbent, Saccharomyces cerevisiae Biomass
Source: Materials (Basel). 2021 Aug 23;14(16):4763. doi: 10.3390/ma14164763 (PMC8400443; doi:10.3390/ma14164763)
Supplement: Supplementary file 1 [file materials-14-04763-s001.zip › materials-1323029-supplementary.pdf]

materials

Supplementary Materials

Treatment of Rhenium-Containing Effluents Using Environmentally Friendly Sorbent, *Saccharomyces cerevisiae* Biomass

Inga Zinicovscaia [1,2,*], Nikita Yushin [1], Dmitrii Grozdov [1], Konstantin Vergel [1], Pavel Nekhoroshkov [1] and Elena Rodlovskaya [3]

1 Department of Nuclear Physics, Joint Institute for Nuclear Research, Joliot-Curie Str., 6, 1419890 Dubna, Russia; ynik_62@mail.ru (N.Y.); dsgrozdov@rambler.ru (D.G.); verkn@mail.ru (K.V.) p.nekhoroshkov@gmail.com (P.N.)

2 Department of Nuclear Physics, Horia Hulubei National Institute for R&D in Physics and Nuclear Engineering, 30 Reactorului Str. MG-6 Magurele, Romania

3 Laboratory for Heterochain Polymers, A.N. Nesmeyanov Institute of Organoelement Compounds of Russian Academy of Sciences, Vavilova Str., 28, 119991 Moscow, Russia; ro745@mail.ru

* Correspondence: zinikovskaia@mail.ru; Tel.: +7-4962165609

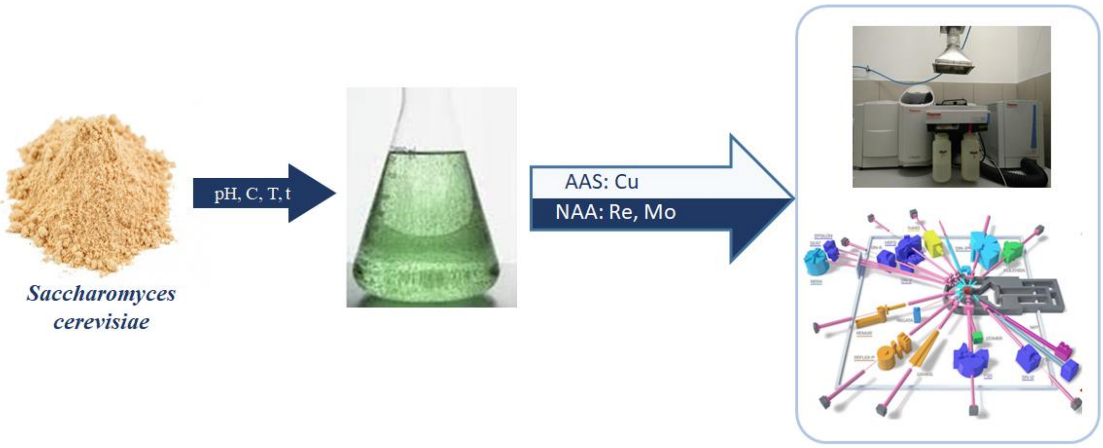

Figure S1. The scheme of biosorption experiment.

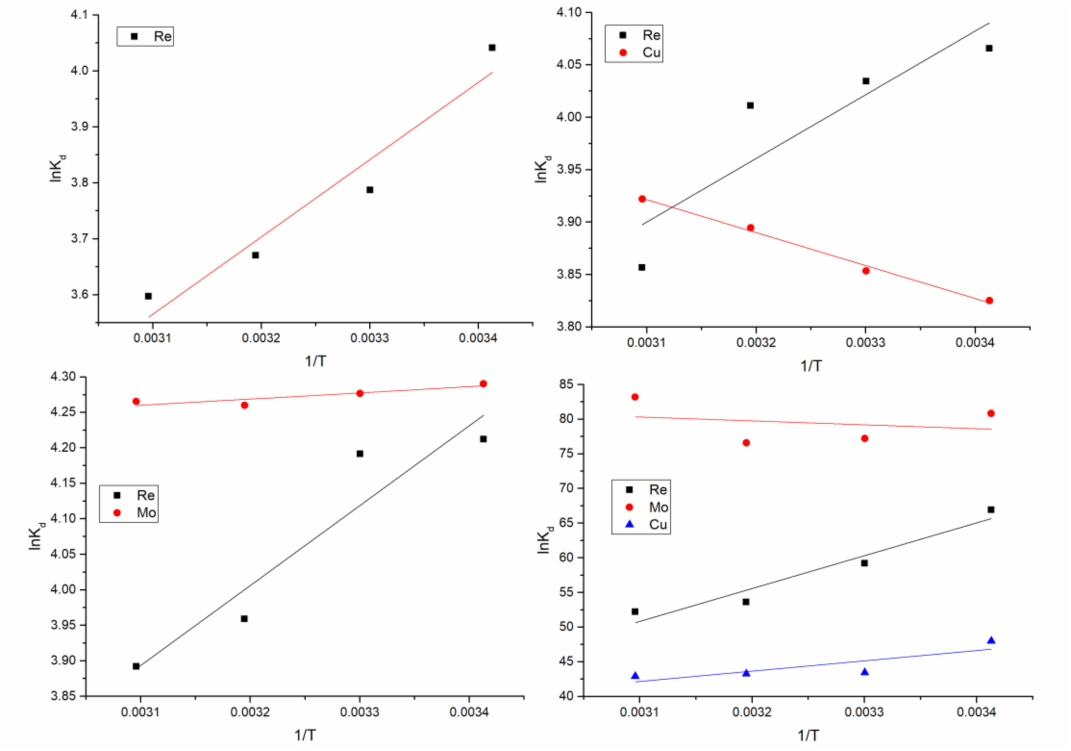

Figure S2. The linear plots of lnKd versus 1/T for the adsorption of metal ions.
